# Two Decades of Molecular Surveillance in Kenya Reveal Shifting *Plasmodium falciparum* Drug Resistance Mutations Linked to Frontline Drug Changes

**DOI:** 10.4269/ajtmh.25-0513

**Published:** 2026-11-06

**Authors:** George A. Tollefson, Benjamin H. Opot, Titus Kipkemboi Maina, Dennis W. Juma, Raphael O. Okoth, Jackline A. Juma, Gladys Chemwor, Edwin W. Mwakio, Abebe A. Fola, John M. Onge’cha, Bernhards R. Ogutu, Rebecca M. Crudale, Jeffrey A. Bailey, Hoseah M. Akala

**Affiliations:** ^1^ Department of Pathology and Laboratory Medicine, Brown University, Providence, Rhode Island, USA;; ^2^ Center for Computational Molecular Biology, Brown University, Providence, Rhode Island, USA;; ^3^ Kenya Medical Research Institute/Walter Reed Army Institute of Research, Africa, Kisumu, Kenya;; ^4^ Kenya Medical Research Institute, Kisumu and Nairobi, Kenya

## Abstract

Malaria control has faced repeated challenges due to the emergence and spread of antimalarial resistance. In the present study, temporal trends in *Plasmodium falciparum* resistance mutations were analyzed using molecular inversion probe sequencing of 625 samples from 11 Kenyan sites (1998–2021), spanning multiple frontline treatments. After the phased adoption of artemether–lumefantrine as first-line treatment of uncomplicated malaria in 2006, a rapid increase in multidrug resistance protein 1 N86 and D1246 alleles associated with reduced lumefantrine susceptibility was observed, along with an accelerated decline in the chloroquine resistance transporter CVIET haplotype associated with chloroquine and amodiaquine resistance. Sulfadoxine–pyrimethamine (SP) resistance mutations (dihydrofolate reductase [DHFR] 51I, 59R, 108N; dihydropteroate synthase 437G, 540E) reached near-fixation after the introduction of SP as the first-line therapy for uncomplicated malaria in 1998 and persisted at near-fixation through 2021. The high-level pyrimethamine resistance mutation DHFR 164L emerged and expanded after 2012. Furthermore, mutations associated with reduced artemisinin efficacy were identified in ubiquitin-specific protease 1, adaptor protein complex 2, mu subunit, and sarcoplasmic/endoplasmic reticulum calcium adenosine triphosphatase. Although no validated kelch13 (K13) artemisinin partial resistance mutations were detected, several novel K13 propeller domain missense mutations, including 473F and 569S, were observed after the adoption of artemisinin-based combination therapies. These findings reveal the rapid, dynamic evolution of drug resistance in the context of shifting antimalarial drug pressures and underscore the need for sustained genomic surveillance in malaria-endemic regions to inform adaptive treatment strategies. The persistence of SP resistance and the emergence of markers associated with reduced susceptibility to artemisinins and partner drugs raise concerns about the long-term efficacy of current antimalarials for treatment, seasonal malaria chemoprevention, and intermittent preventive interventions.

## INTRODUCTION

Malaria is a life-threatening disease caused by protozoan parasites of the genus *Plasmodium*, with *Plasmodium falciparum* being the most virulent species and the leading cause of severe disease and death, particularly in sub-Saharan Africa. In 2024, an estimated 610,000 deaths and 282 million malaria cases occurred globally, with 4.2 million cases occurring in Kenya.^1^ Artemisinin-based combination therapies (ACTs) remain the cornerstone of *P. falciparum* malaria treatment worldwide. Artemisinin-based combination therapies combine a powerful, short-acting artemisinin-derived compound with a long-acting partner drug, most commonly lumefantrine (LU), piperaquine (PPQ), or amodiaquine (AQ) in sub-Saharan Africa. However, progress toward global malaria control is increasingly threatened by the evolution of *P. falciparum* resistance to frontline antimalarials. Historically, emerging drug resistance in Africa was characterized by the importation of resistant lineages from Southeast Asia.^2^^,^^3^ However, the recent independent emergence of kelch13 (K13) mutations associated with frontline drug artemisinin partial resistance (ART-R) in East Africa and the Horn of Africa signals a critical shift in the drug-resistance landscape of the continent.^4^^–^^10^

Kenya’s antimalarial treatment policy has evolved significantly over the past two decades, driven by declining drug efficacy, updated recommendations from regional and global health organizations, and a national commitment to expanding access to efficacious therapies (Figure 1A). Although chloroquine (CQ) was officially replaced by sulfadoxine–pyrimethamine (SP) as the first-line therapy for uncomplicated malaria in 1998 because of widespread CQ treatment failure,^11^ the transition was characterized by a delayed and incomplete rollout. Between 1998 and 2005, SP implementation faced significant hurdles. As late as 2002, retailer stocks of SP remained low (28.5%) compared with 93.4% for second-line antimalarial AQ and 15.1% for CQ.^12^ The incomplete implementation of SP was driven by legal barriers, a lack of national communication strategies for the private sector, and the continued popularity of AQ and CQ, which remained widely available through 2005 despite policy changes.^13^ Consequently, during this transition period (1998–2005), *P. falciparum* parasites in Kenya were subjected to a heterogeneous mix of drug pressures from SP, AQ, quinine, and residual CQ use. Despite this incomplete widespread adoption of SP in Kenya, by mid-June 2001, studies revealed SP failure rates in excess of 25%.^14^^,^^15^

**Figure 1.**
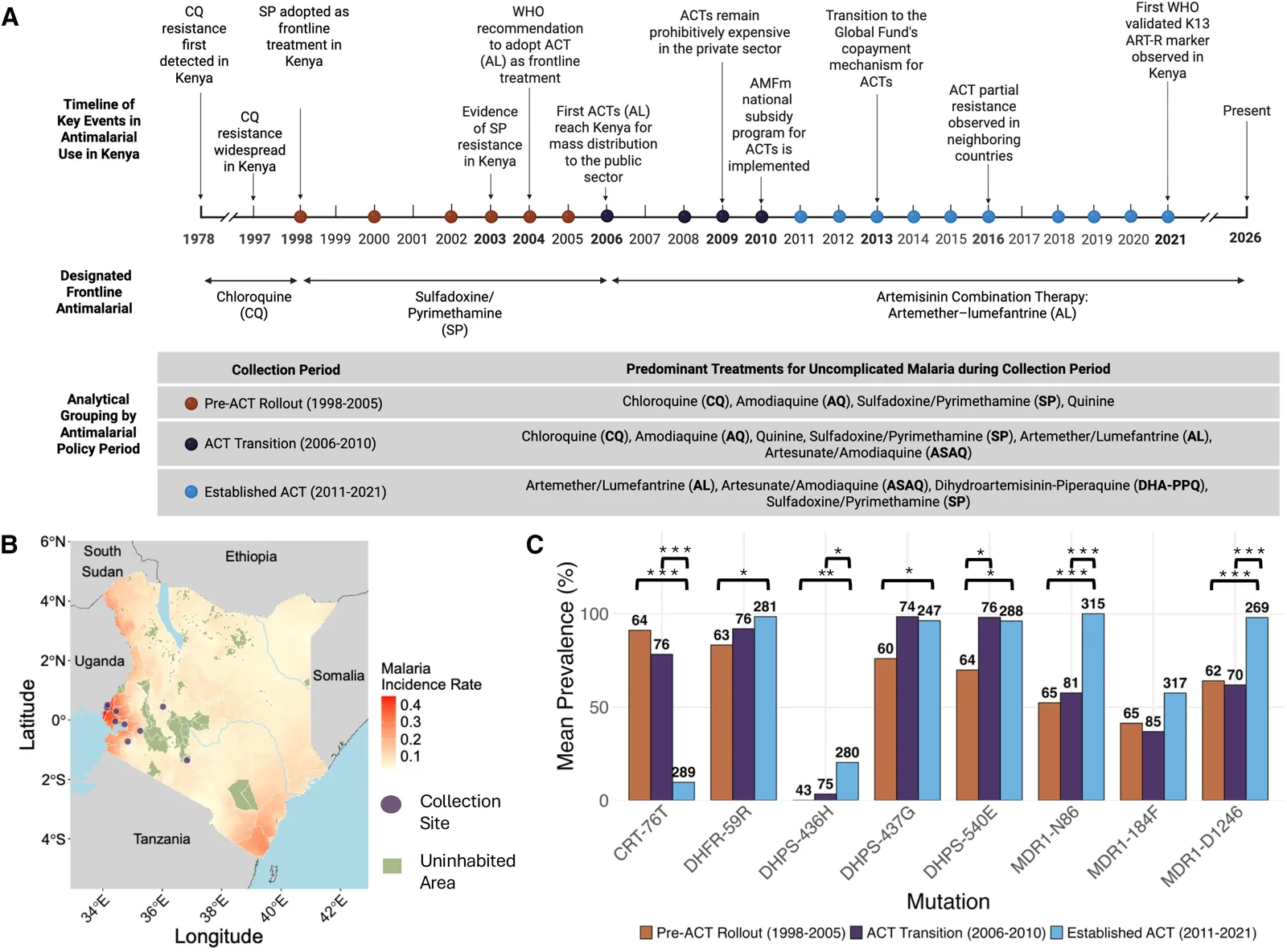
Timeline and locations of sample collection across varying malaria transmission settings spanning changing mutation frequencies in the context of changing frontline antimalarials. (**A**) Timeline of sample collection years relative to national antimalarial policy transitions and the emergence of *Plasmodium falciparum* resistance markers in Kenya. (**B**) Map of collection sites indicated by purple points overlaid on a geospatial choropleth map of malaria incidence rate (mean cases per 1,000 population, 2000–2021; Malaria Atlas Project). The temporal decline in yearly mean incidence across these sites during the study period is summarized in Supplemental Figure 1. (**C**) Mean prevalence of key shifting drug-resistance mutations in *P. falciparum* across three defined antimalarial policy eras: Pre-artemisinin-based combination therapy (ACT) rollout (1998–2005; *n* = 66), ACT transition (2006–2010; *n* = 104), and established ACT (2011–2021; *n* = 455). Bars represent the mean prevalence (%) of each mutation within its respective era. The number of successfully genotyped samples at each evaluated locus (unique molecular identifiers ≥5) is indicated on top of each bar. Statistical significance for pairwise comparisons between eras was assessed using Wilcoxon rank-sum tests with Holm-Bonferroni adjustment. Significance levels are indicated as follows: * *P*_adj_ <0.001; † *P*_adj_ <0.01; ‡ *P*_adj_ <0.05.

A second critical shift in Kenyan antimalarial treatment policy occurred with the adoption of the ACT, artemether–LU (AL), as the first-line treatment of uncomplicated malaria in 2006. Although AL was designated as the frontline treatment in 2004, logistical hurdles delayed its arrival to Kenya until 2006, and its initial use was restricted to the public sector to build safety profiles and public trust.^13^ Between 2006 and 2010, the implementation of ACTs was challenged by frequent public-sector shortages and high private-sector prices, which reached upwards of $6.00 per dose.^13^^,^^16^^,^^17^ Surveys during this time indicated that 75% of public-sector health facilities lacked at least one of four weight-specific packs of AL; consequently, ∼25% of patients were still treated with quinine, AQ, or SP.^16^^,^^18^ It was not until the 2010–2012 implementation of the Affordable Medicines Facility, Malaria (AMFm), Global Fund subsidy pilot, that AL became both affordable ($0.50 per dose) and widely available in the Kenyan private sector, where availability rose from 21% to more than 60%.^17^ Since 2013, this accessibility has been sustained through the Global Fund co-payment mechanism.^19^ In 2015, the availability of quality-assured ACTs (QAACTs) reached 70%, and by 2020, the Kenya Malaria Indicator Survey revealed that 82% of children with uncomplicated malaria received AL.^19^^,^^20^

Although AL remains the first-line treatment for uncomplicated *P. falciparum* malaria in 2026, SP continues as a standard of care for intermittent preventive treatment in pregnancy (IPTp)^1^ and for expanded use in combination with AQ (SPAQ) for seasonal malaria chemoprevention (SMC). After the successful 2024 pilot in Turkana County, SMC has been expanded to include additional semi-arid seasonal transmission zones in Kenya,^21^ including Marsabit and West Pokot, to protect children during peak transmission seasons. These sustained shifts in antimalarial deployment have reshaped selective pressures on *P. falciparum*, underscoring the need for genomic surveillance to monitor adaptive evolution.

Genomic surveillance of *P. falciparum* involves the use of specific molecular markers to indicate shifts in drug efficacy. Artemisinin partial resistance is primarily mediated by nonsynonymous mutations in the K13 propeller domain. The WHO categorizes these propeller domain mutations as either validated markers, those with proven clinical associations with delayed parasite clearance and confirmed in vitro reduced susceptibility, or candidate markers, which exhibit preliminary evidence of resistance but lack definitive clinical and laboratory confirmation.^22^

Although still adequately efficacious, reduced AL treatment efficacy was first reported in Kenya in 2011, though these early observations were likely influenced by diminished host immunity in coastal regions due to reductions in malaria transmission.^23^ By 2016, validated K13 mutations were spreading in multiple neighboring countries,^4^^–^^8^^,^^24^^–^^26^ and in 2024, low-frequency validated ART-R associated K13 mutations 469Y, 561H, and 675V were observed in Kenyan samples collected in 2021.^9^^,^^27^^,^^28^

Resistance to 4-aminoquinolines (CQ and AQ) and the widely used ACT partner drug LU is primarily mediated by polymorphisms in the digestive vacuole transporters CQ resistance transporter (CRT) and multidrug resistance protein 1 (MDR1). Although CRT 76T is the primary mediator of CQ resistance,^29^ the MDR1 86Y mutation increases CQ resistance and reduces susceptibility to AQ.^30^ In contrast, LU, the partner drug in AL, selects the opposite profile, favoring the wild-type alleles (CRT K76 and MDR1 N86).^31^^,^^32^ Artemether–LU deployment has also been associated with increases in the MDR1 N86-184F-D1246 (NFD) haplotype.^33^ Although MDR1 copy number amplifications are associated with reduced LU and mefloquine susceptibility in Southeast Asia,^34^ these mutations remain rare in Africa,^35^ where mefloquine is not commonly deployed.^1^ However, no validated molecular marker of clinical LU resistance has been established.

Antifolate resistance to SP arises through stepwise accumulation of mutations in dihydrofolate reductase (DHFR) and dihydropteroate synthase (DHPS), which reduce susceptibility to pyrimethamine and sulfadoxine, respectively.^36^ These cumulative mutations have led to widespread reduced clinical efficacy of SP across sub-Saharan Africa^37^ due to the near-fixation of the triple mutant haplotype DHFR IRN (51I-59R-108N) and the double mutant haplotype DHPS GE (437G-540E).^38^^,^^39^ New highly resistant alleles in each protein (DHFR 164L and DHPS 581G), comprising DHFR INRL and DHPS GEG mutant haplotypes, now further threaten SP-based chemoprevention strategies such as IPTp and SMC.^40^^,^^41^

Despite the public health importance of monitoring these markers, most molecular surveillance efforts have been temporally fragmented, limiting understanding of how successive shifts in antimalarial policy have shaped the genomic resistance profiles of Kenyan *P. falciparum* populations. Here, the authors address this gap by successfully genotyping 625 *P. falciparum* infections collected across a 23-year period (1998–2021) in Kenya. Using a panel of 815 molecular inversion probes (MIPs) targeting 14 key *P. falciparum* drug resistance genes, they provide a continuous characterization of genetic changes as Kenya transitioned through multiple frontline treatments.

## MATERIALS AND METHODS

### Sample collection.

A total of 719 archived peripheral blood DNA samples were sequenced for the present study. Samples were collected between 1998 and 2021 from clinical malaria patients over 6 months of age living near 11 collection sites primarily located in Western Kenya (Figure 1A and B).^42^^–^^44^ Although mean malaria incidence across these sites declined sharply from ∼0.6 to 0.2 cases per 1,000 population between 2002 and 2006, transmission remained relatively stable and low (∼0.13 to 0.2 cases per 1,000) for the remainder of the study period through 2021 (Supplemental Figure 1). Throughout the entire study timeframe, the sampled sites consistently represented high-burden regions of Kenya relative to the rest of the country (Supplemental Figure 2). Collection sites included outpatient departments at hospitals in Nairobi, Kisumu, Alupe, Mumias, Kombewa, Kericho, Kisii, Marigat, Busia, Entasopia, and Isiolo. Samples were collected from patients with clinical malaria confirmed via microscopy or rapid diagnostic test according to site-specific protocols. The number of samples successfully sequenced each year is reported in Supplemental Table 1.

### Antimalarial policy era classification.

To statistically evaluate genomic shifts in the context of changing drug pressures and real-world drug utilization, samples were grouped a priori into three eras on the basis of the antimalarial treatment policy milestones and drug availability shifts detailed in the background (Figure 1A). The pre-ACT rollout period (1998–2005; *n* = 66) represents the period after the official transition from CQ to SP as first-line therapy for uncomplicated malaria in 1998, which was characterized by the continued widespread use of legacy 4-aminoquinolines, including CQ and AQ, as well as oral quinine alongside SP. The dataset for this era includes 14 samples documented by collection sites as “Pre-ACT” without a specific year of collection. These were included in era-wide prevalence reporting and statistical comparisons because they were confirmed to predate 2006, but they were excluded from year-specific analyses. The ACT transition period (2006–2010; *n* = 104) encompasses the public sector introduction of AL and the continued use of legacy drugs CQ, AQ, SP, and quinine, as well as alternative ACTs, such as artesunate–AQ (ASAQ) and dihydroartemisinin–PPQ. Finally, the established ACT period (2011–2021; *n* = 455) represents the large-scale expansion of QAACTs, primarily AL, after the 2010 national AMFm subsidy pilot and subsequent formalization of the Global Fund copayment mechanism in 2013, which has sustained the availability and affordability of QAACTs to date. This classification framework reflects the persistent selective pressure of legacy drugs and the protracted nature of policy transitions, allowing for a more accurate delineation of the evolutionary trajectory of the Kenyan *P. falciparum* population over two decades.

### Molecular inversion probe sequencing of drug resistance loci.

Total genomic DNA was extracted from whole blood using the Qiagen DNA Mini protocol (Qiagen, Hilden, Germany) and eluted in 150 *µ*L of buffer. *Plasmodium* genus and species identification was performed via a two-step quantitative real-time polymerase chain reaction (qPCR) test targeting the 18S ribosomal RNA on the Applied Biosystems QuantStudio 6 Flex (Thermo Fisher Scientific, Waltham, MA, USA). Samples confirmed as *P. falciparum* with a cycle threshold value ≤31 were selected for MIP sequencing.^45^^–^^48^

Extracted DNA was captured with MIPs using the DR2 MIP panel^49^ consisting of 815 probes targeting 14 known *P. falciparum* antimalarial drug resistance genes encoding proteins ubiquitin-specific protease 1 (UBP1), adaptor protein complex 2, mu subunit (AP2MU), sarcoplasmic/endoplasmic reticulum calcium adenosine triphosphatase (ATP6), apicoplast ribosomal protein S10, CRT, cytochrome b, DHFR, DHPS, ferredoxin, K13, MDR1, multidrug resistance protein 2, phosphoinositide-binding protein 7, and protein phosphatase. Molecular inversion probe capture, library preparation, and 150-base pair paired-end sequencing on an Illumina NextSeq550 (Illumina, San Diego, CA, USA) instrument were conducted at Brown University (Providence, Rhode Island, USA) as previously described.^49^

### Sequence data analysis and drug resistance prevalence estimation.

Sequence data were processed and initially analyzed with the MIPTools pipeline v0.5.0 (https://github.com/bailey-lab/MIPTools), which encapsulates a number of key programs and steps.^49^ In brief, MIPWrangler (https://github.com/bailey-lab/MIPWrangler) was used to stitch paired-end reads, remove sequencing errors using unique molecular identifiers (UMIs; random oligonucleotide sequences that tag individual DNA templates before amplification), and construct target consensus sequences. Raw reads were aligned to the *P. falciparum* 3D7 reference genome (PlasmoDB-42_Pfalciparum3D7 obtained from https://plasmodb.org/plasmo/app) using the Burrows-Wheeler Aligner BWA-MEM algorithm v0.7.17.^50^ Freebayes 1.3.6 was used to call variants.^51^

Downstream analyses were performed using variant call format files, which were cross-referenced with the 3D7 transcriptome to identify coding mutations and generate genotype tables. To ensure high-confidence calling, variants were quality-filtered by removing any locus within a sample supported by fewer than five UMIs. These data were used to calculate the prevalence of known drug resistance-associated mutations in genes encoding proteins including K13, CRT, MDR1, DHPS, DHFR, AP2MU, UBP1, ATP6, and others associated with drug resistance.

Prevalence was defined as *p = m/n* × 100% where *m* is the number of samples carrying a variant allele, and *n* is the number of samples with a called genotype at the locus. Single-marker prevalence calculations included both homozygous and heterozygous genotypes, defining prevalence as the proportion of individuals carrying the mutant allele.

The prevalence of multilocus haplotypes was calculated similarly (*p = m*/*n* × 100%, where *m* is the number of samples carrying each haplotype and *n* is the number of samples with genotypes called at all haplotype alleles). For single-gene multilocus haplotype prevalence reporting, a dominant-clone approach was used to maximize data retention while ensuring linkage accuracy. Samples were included in single-gene haplotype analysis only if a predominant allele reached a within-sample allele frequency ≥80% with UMI ≥5 coverage at every targeted locus in the given gene. Multilocus haplotypes were then constructed using these dominant alleles, ensuring a high-confidence representation of the major parasite strain within each infection. Individual allele and haplotype prevalence estimations were visualized using the R package ggplot2 (v3.4.0).

### Spatiotemporal and transmission analysis.

Temporal trends were visualized using 2-year moving averages to mitigate the impact of low-density sampling in specific intervals (e.g., 1998–1999, *n* = 4; 2006–2007, *n* = 1), providing a more stable representation of longitudinal shifts in allele prevalence. Samples lacking a specific year of collection (the 14 “Pre-ACT” samples mentioned above) were excluded from these finer-scale temporal analyses but remained in the era-based statistical tests.

To contextualize these genomic shifts within the broader landscape of Kenyan malaria control, regional transmission intensity was evaluated. Administrative boundaries and annual mean parasite incidence rasters were obtained from the Malaria Atlas Project, and incidence rates for the study sites were extracted to assess longitudinal shifts in transmission intensity (Supplemental Figures 1 and 2).

## STATISTICAL ANALYSES

All statistical analyses were conducted using R (v4.2.2; R Foundation, Vienna, Austria). To examine the association between shifting drug policies and *P. falciparum* genomic signatures of resistance, statistical differences in prevalence were assessed using Wilcoxon rank-sum tests. To account for multiple comparisons, *P*-values were adjusted using the Holm-Bonferroni method, with a significance threshold of *P*_adj_ <0.05.

## RESULTS

### Lack of candidate or validated ART-R markers.

Among the 625/719 (86.9%) samples successfully sequenced, the authors first confirmed that no WHO-validated or candidate K13 mutations associated with ART-R were detected in this dataset during the study period. However, novel K13 propeller domain missense mutations of unknown significance were detected after the implementation of ACTs (Supplemental Table 2). The K13 569S variant was detected in one sample from Kisii in 2012, and the 473F variant was found in a single sample from Marigat in 2020. K13 578S, a previously reported polymorphism unassociated with ART-R and common throughout sub-Saharan Africa, was detected sporadically (1% to 8.8%) in various sites in Western Kenya throughout the entire study period in 2000, 2008, 2009, 2014, 2015, 2016, 2019, and 2020 (Supplemental Figure 3; Supplemental Table 2).

### Summary of major changes in antimalarial resistance markers across treatment eras.

The prevalence of MDR1, CRT, DHFR, and DHPS mutations shifted significantly across the three study periods (Figure 1C; Supplemental Tables 3–6). Mutations associated with CQ resistance declined substantially by the established ACT period (beginning 2011). Specifically, CRT 76T decreased significantly from 91.1% during the pre-ACT rollout (1998–2005) period to 9.7% in the established ACT (2011–2021) period (*P*_adj_ = 9.6 × 10^−7^). Conversely, markers associated with SP resistance either remained near fixation or increased, with DHFR 59R, DHPS 437G, and DHPS 540E all exceeding 96% prevalence by the established ACT period. Notably, the DHPS 436H mutation emerged during the ACT transition period and increased significantly to 20.3% by the established ACT period (*P*_adj_ = 4.68 × 10^−3^). The prevalence of MDR1 alleles associated with reduced LU susceptibility also increased significantly, coinciding with widespread AL uptake. The wild-type MDR1 N86 allele rose from 52.3% in the pre-ACT rollout period to near-fixation at 99.9% in the established ACT period (*P*_adj_ = 6.45 × 10^−9^), and MDR1 D1246 increased from 64.0% to 97.9% (*P*_adj_ = 3.52 × 10^−5^).

### Increasing prevalence of the MDR1 NFD haplotype associated with reduced susceptibility to LU.

After 2010, the MDR1 N86 and D1246 alleles rose quickly to near-fixation (Figure 2A; Supplemental Table 3). Haplotype analysis revealed a transition from high prevalence of the AQ- and CQ-resistance-associated MDR1 86Y-Y184-1246Y (YYY) haplotype to the NFD and NYD haplotypes, both carrying key N86 and D1246 alleles associated with reduced susceptibility to LU and sensitivity to 4-aminoquinolines CQ and AQ by 2011. These haplotypes reached prevalence rates of 39.5% and 58.1%, respectively, by 2018–2021 (Figure 2B). Temporal analysis confirmed that although wild-type N86 and D1246 alleles existed at moderate frequency (<50%) during the pre-ACT rollout period before 2006, they rapidly increased in frequency after the transition to ACTs (Figure 2B and C).

**Figure 2.**
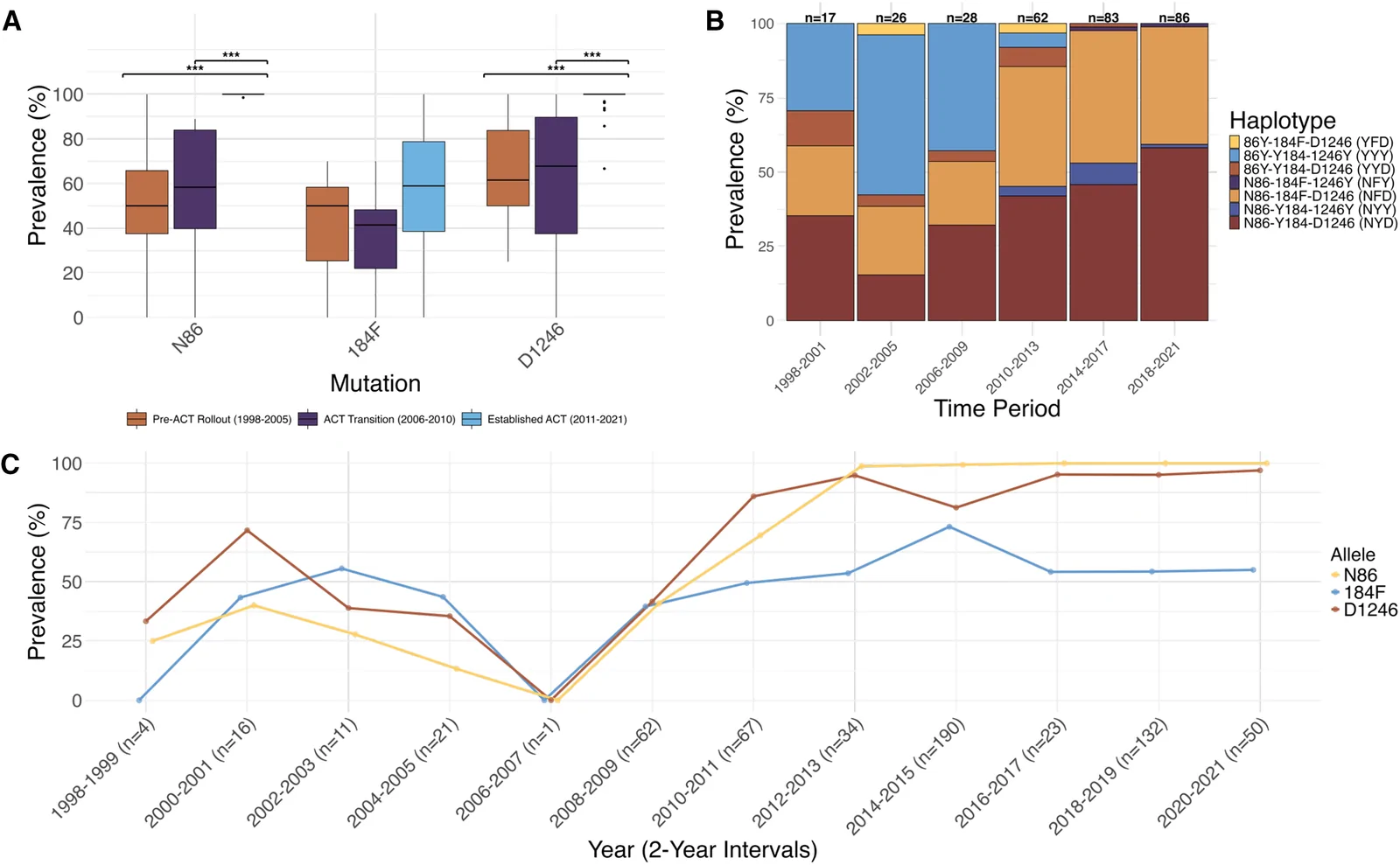
Frequency of key multidrug resistance protein 1 (MDR1) alleles associated with reduced susceptibility to lumefantrine. (**A**) Prevalence of key MDR1 alleles across three antimalarial policy eras: pre-artemisinin-based combination therapy (ACT) rollout (brown; 1998–2005), ACT transition (purple; 2006–2010), and established ACT (blue; 2011–2021). Boxplots represent the distribution of annual allele prevalences calculated for each site-year collection within each era. Wilcoxon-derived Holm-Bonferroni adjusted *P*-values are indicated above comparisons (* *P*_adj_ <0.001; † *P*_adj_ <0.01; ‡ *P*_adj_ <0.05). (**B**) Temporal trends in the prevalence of within-sample dominant MDR1 haplotypes. Sample sizes (*n*) after filtering for dominant haplotype analysis are indicated above the stacked bars for each time point. (**C**) Temporal trends in individual allele prevalences are shown as 2-year moving averages. Annual sample sizes for the total dataset are provided in the *x*-axis labels.

### Rapid reversion to CQ sensitivity after CQ withdrawal and ACT implementation.

Chloroquine resistance transporter mutations associated with CQ resistance remained predominant during the pre-ACT rollout and the ACT transition period but began to decline sharply by the end of the ACT transition period in 2010, ultimately reaching low prevalence by 2012–2013 (Figure 3A–C). Key alleles, including CRT 74I, 76T, and 220S, transitioned from near-fixation to significantly lower levels (<10%) during the established ACT period (*P*_adj_ <0.001; Supplemental Table 4). Haplotype analysis revealed the replacement of dominant AQ- and CQ-resistant C72-v. 73-74I-75E-76T-220S-326S (CVIETSS) and C72-v. 73-74I-75E-76T-220S-N326 (CVIETSN) haplotypes with the wild-type C72-v. 73-M74-N75-K76-A220-N326 (CVMNKAN) haplotype (Figure 3B) by 2013. Despite the overall decline, a modest resurgence of parasites carrying the CQ resistance-associated CVIETS haplotype occurred between 2018 and 2021 (Figure 3C).

**Figure 3.**
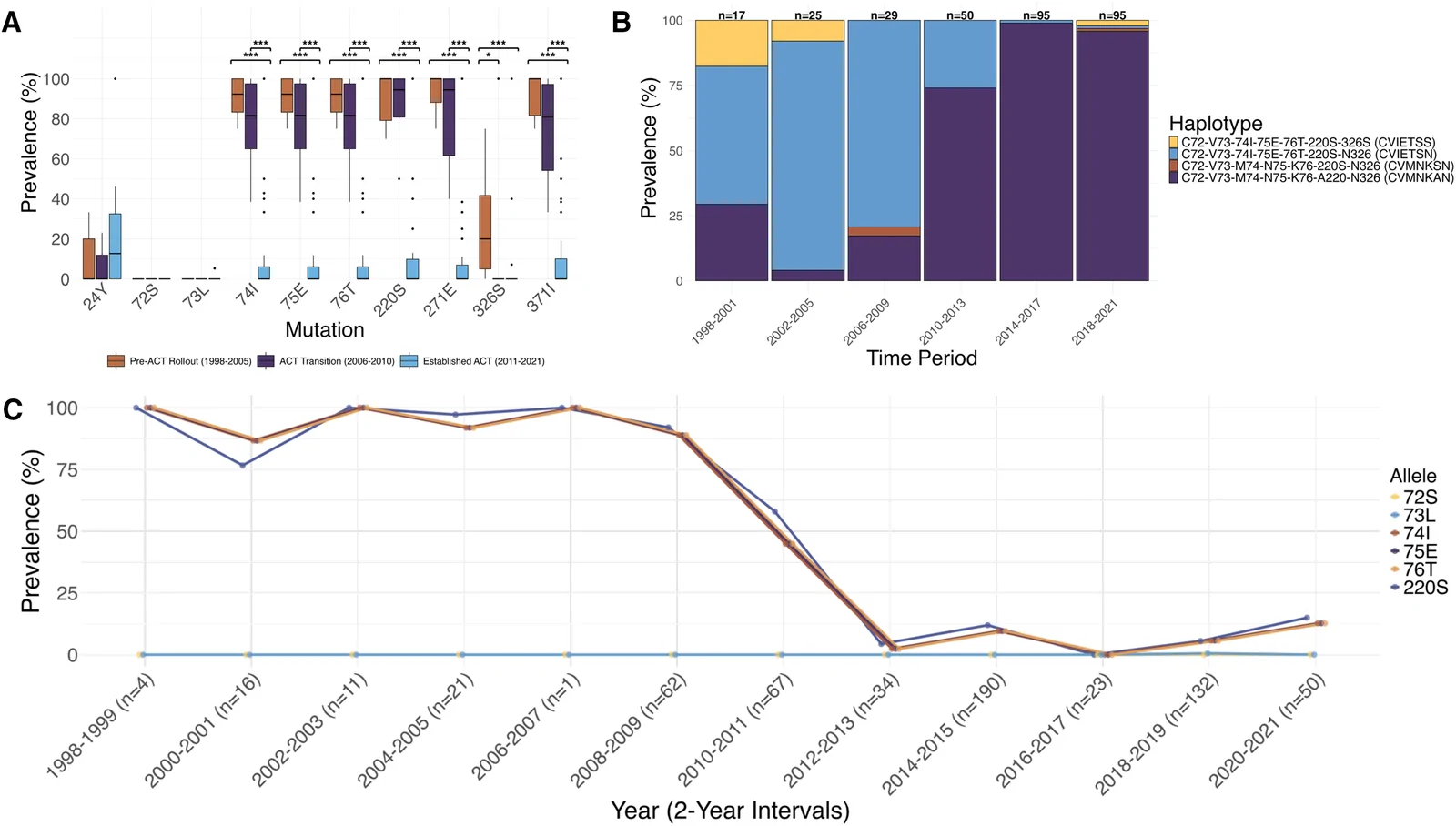
Rapid reversion to chloroquine sensitivity after artemisinin-based combination therapy (ACT) implementation. (**A**) Prevalence of key chloroquine resistance transporter (CRT) alleles across three antimalarial policy eras: pre-ACT rollout (brown; 1998–2005), ACT transition (purple; 2006–2010), and established ACT (blue; 2011–2021). Boxplots represent the distribution of annual allele prevalences calculated for each site-year collection within each era. Wilcoxon-derived Holm-Bonferroni adjusted *P*-values are indicated above comparisons (* *P*_adj_ <0.001; † *P*_adj_ <0.01; ‡ *P*_adj_ <0.05). (**B**) Temporal trends in the prevalence of within-sample dominant CRT haplotypes. Sample sizes (*n*) after filtering for dominant haplotype analysis are indicated above the stacked bars for each time point. (**C**) Temporal trends in individual allele prevalences are shown as 2-year moving averages. Annual sample sizes for the total dataset are provided in the *x*-axis labels.

### Sustained DHFR and DHPS mutations associated with SP resistance at near-fixation 20 years after SP implementation.

In contrast to the reversion seen in CRT wild-type alleles after CQ withdrawal, markers of SP resistance remained at high prevalence or increased across all periods. In DHFR, the triple-mutant 51I-59R-108N (IRN) haplotype was dominant by 2002, 4 years after implementation of SP as frontline antimalarial, and remained at high prevalence, reaching near-fixation by the start of the ACT transition period in 2006 (Figure 4A and B). The 164L mutation, which is associated with high-level resistance, emerged midway through the ACT transition period in 2008–2009 (5%) and increased modestly in the final years of the study (Supplemental Table 5), reaching 15.2% in 2020–2021. Although it was initially observed on the 51I-C59-108N background, 164L was observed as part of the quadruple DHFR 51I-59R-108N-164L (IRNL) haplotype by the end of the study.

**Figure 4.**
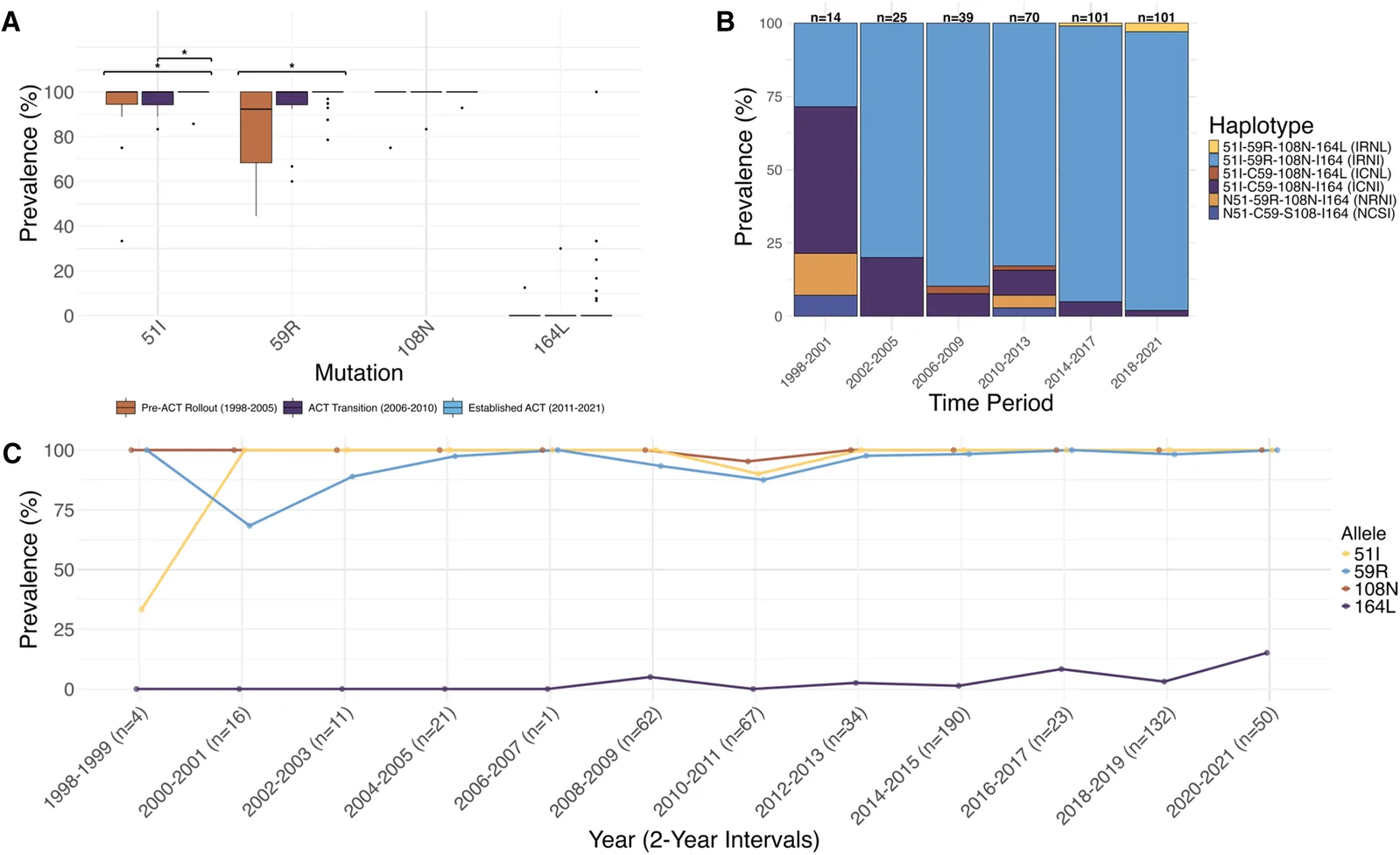
Sustained near-fixation of dihydrofolate reductase (DHFR) mutations associated with decreased susceptibility to pyrimethamine. (**A**) Prevalence of key DHFR alleles across three antimalarial policy eras: pre-artemisinin-based combination therapy (ACT) rollout (brown; 1998–2005), ACT transition (purple; 2006–2010), and established ACT (blue; 2011–2021). Boxplots represent the distribution of annual allele prevalences calculated for each site-year collection within each era. Wilcoxon-derived Holm-Bonferroni adjusted *P*-values are indicated above comparisons (* *P*_adj_ <0.001; † *P*_adj_ <0.01; ‡ *P*_adj_ <0.05). (**B**) Temporal trends in the prevalence of within-sample dominant DHFR haplotypes. Sample sizes (*n*) after filtering for dominant haplotype analysis are indicated above the stacked bars for each time point. (**C**) Temporal trends in individual allele prevalences are shown as 2-year moving averages. Annual sample sizes for the total dataset are provided in the *x*-axis labels.

For DHPS, significant shifts in alleles were observed, including the near-elimination of 436A and 613S by the established ACT period (Figure 5A; Supplemental Table 6). Conversely, the DHPS 437G-540E (GE) haplotype remained dominant (Figure 5B), and the 436H mutation increased significantly from 0% during the pre-ACT rollout period to >20% by the end of the study (*P*_adj_ <0.01).

**Figure 5.**
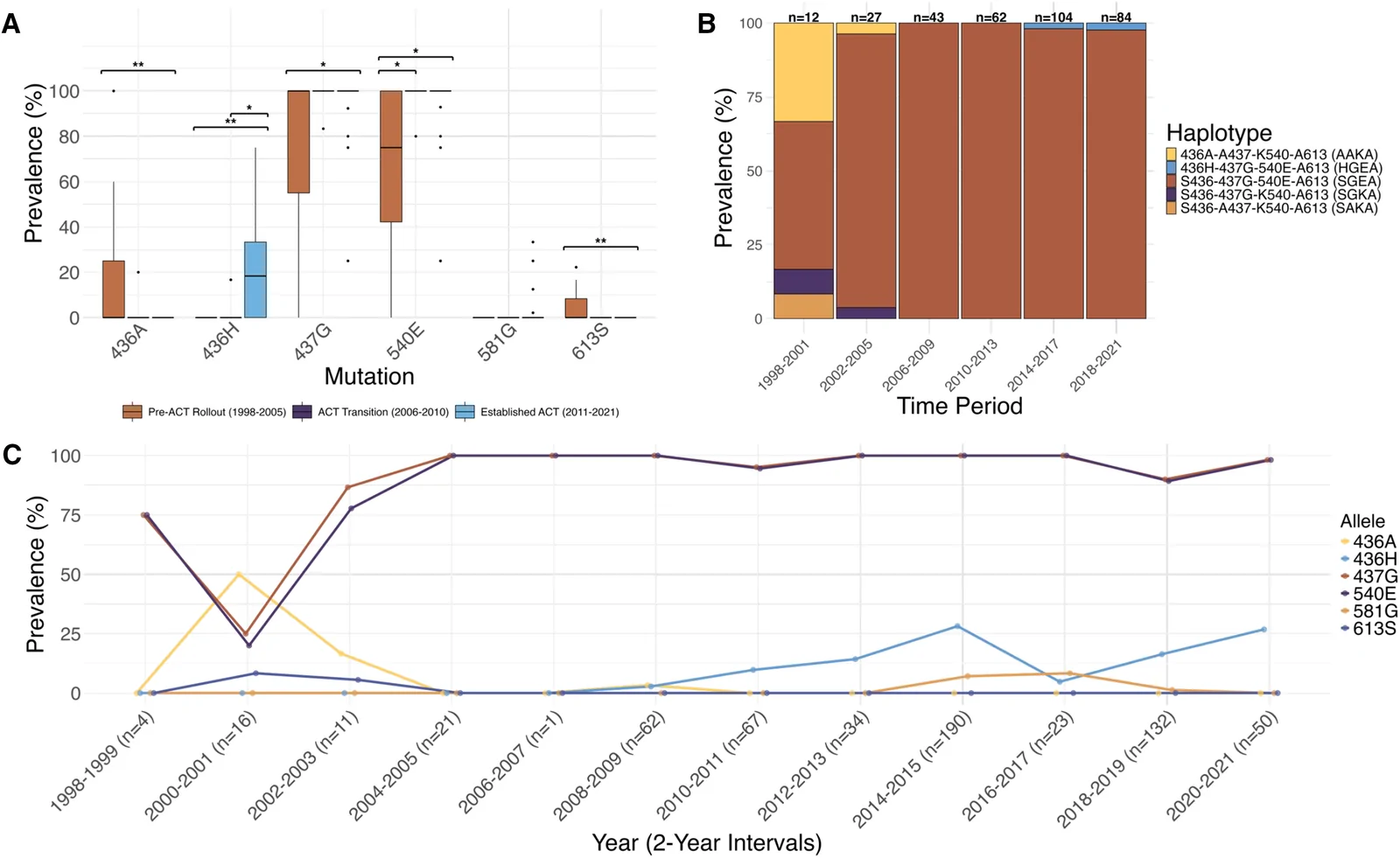
Sustained near-fixation of dihydropteroate synthase (DHPS) mutations associated with decreased susceptibility to sulfadoxine. (**A**) Prevalence of key DHPS alleles across three antimalarial policy eras: pre-artemisinin-based combination therapy (ACT) rollout (brown; 1998–2005), ACT transition (purple; 2006–2010), and established ACT (blue; 2011–2021). Boxplots represent the distribution of annual allele prevalences calculated for each site-year collection within each era. Wilcoxon-derived Holm-Bonferroni-adjusted *P*-values are indicated above comparisons (* *P*_adj_ <0.001; † *P*_adj_ <0.01; ‡ *P*_adj_ <0.05). (**B**) Temporal trends in the prevalence of within-sample dominant DHPS haplotypes. Sample sizes (*n*) after filtering for dominant haplotype analysis are indicated above the stacked bars for each time point. (**C**) Temporal trends in individual allele prevalences are shown as 2-year moving averages. Annual sample sizes for the total dataset are provided in the *x*-axis labels.

### Temporal trends in mutations associated with artemisinin tolerance.

Dynamic changes were observed in the prevalence of mutations associated with artemisinin tolerance in UBP1, AP2MU, and ATP6 (Supplemental Table 2). Ubiquitin-specific protease 1 1525E rose sharply during the ACT transition before declining and subsequently rebounding during the established ACT period. Similar sporadic patterns were observed for AP2MU 160N and ATP6 431K.

## DISCUSSION

In the present study, retrospective samples spanning more than two decades were sequenced to evaluate long-term temporal trends in drug resistance mutations across several shifts in Kenya’s frontline antimalarial policies. The observed trends broadly align with those observed throughout East Africa. Selective pressures associated with ACTs, SP, and other antimalarials continue to be associated with changes in drug resistance allele prevalence, with important implications for treatment efficacy and future therapeutic strategies.

The persistence of hallmark CQ resistance CRT mutations near fixation 8 years after the replacement of CQ with SP, and the subsequent sharp decline in prevalence after AL implementation, strengthens the hypothesis that CRT wild-type alleles are associated with decreased LU sensitivity. However, the study findings reveal that a near-complete reversion to the CRT K76 wild-type allele did not occur until after the onset of the established ACT period in 2011. The 2010 AMFm national subsidy program and subsequent Global Fund copayment mechanism for private-sector QAACTs likely facilitated the true population-level cessation of 4-aminoquinoline pressure maintaining mutant CRT parasites at high prevalence. Interestingly, a modest resurgence of CRT 76T was observed after 2018. Although this may suggest ongoing drug selection by AQ pressure through private-sector use of ASAQ, the small sample size suggests that it may also be due to parasite importation from border regions, possibly Ethiopia, where CQ remains the frontline treatment for *Plasmodium vivax* malaria and where CRT 76T prevalence remains high.^52^

After the transition to AL in 2006, rapid shifts were observed in alleles associated with decreased sensitivity to LU, specifically the expansion of the MDR1 N86-184F-D1246 (NFD) and N86-Y184-D1246 (NYD) haplotypes, as well as the decline of the MDR1 86Y-Y184-1246Y (YYY) haplotype, which is traditionally associated with resistance to AQ and CQ. Meta-analyses of clinical outcomes and gene-editing studies have revealed that codons 86 and 1,246 mediate LU IC_50_ values,^53^ whereas altering codon 184 has a negligible effect.^54^ The slow pace of the reversion of MDR1 86Y and 1246Y to wild-type N86 and D1246 between 2006 and 2010 likely reflects the reciprocal selection pressures of the ACT transition era, with AL selecting for NFD and NYD haplotypes and AQ selecting for YYY haplotypes.

The opposing selective pressures exerted by ACT partner drugs on MDR1 drug-resistance haplotypes offer a recognized opportunity to mitigate resistance development.^55^^,^^56^ Although AL strongly selects for the MDR1 N86 and D1246 wild-type alleles, AQ, an actively used component of alternative ACT ASAQ and SMC drug SPAQ, exerts the opposite pressure, selecting for the 86Y and 1246Y mutations. This suggests that triple-ACTs (TACTs) combining LU with AQ or other partner drugs could help maintain drug efficacy. Given promising preliminary results from Kenya in 2021,^57^ expanded clinical trials aimed at evaluating AL-AQ or other TACT combinations in East Africa should be prioritized.

In contrast to the dynamic shifts seen in 4-aminoquinoline and ACT efficacy-related markers, SP resistance haplotypes have remained remarkably stable. The triple DHFR IRN and double DHPS GE haplotypes reached fixation shortly after 1998 and have persisted at near-fixation despite SP’s withdrawal from routine case management in 2006, suggesting minimal fitness costs and ongoing pressure from IPTp. More concerning is the emergence of additional resistant haplotypes, including the quadruple DHFR IRNL haplotype carrying 164L and the triple DHPS GEG haplotype carrying 581G, which further threaten SP-based chemoprevention. Given their persistence, the long-term durability of SP for IPTp is increasingly tenuous, underscoring the need to continue monitoring clinical efficacy and investigating alternative preventative treatments.

Although WHO-validated ART-R K13 mutations were not detected in this specific dataset, the study collection concluded in 2021, which was the pivotal year in which *P. falciparum* carrying validated ART-R K13 mutations were first confirmed in Kenya. Since then, validated K13 mutations 469Y, 561H, and 675V have been documented in Kenyan parasites, marking a critical shift in the country’s drug resistance landscape. In addition to novel, uncharacterized K13 propeller mutations, nonsynonymous variants in UBP1 (1525E), AP2MU (160N), and ATP6 (431K) that are increasingly recognized for their potential role in modulating parasite susceptibility to artemisinins were identified.^58^^,^^59^

Beyond direct drug selection, the authors considered the role of broader epidemiological shifts in the context of changing drug resistance mutation profiles. Although mean *P. falciparum* malaria incidence across the study sites fell by ∼66% between 2002 and 2006 to ∼0.2 cases per 1,000 population, transmission remained relatively stable and low for the subsequent 15 years (Supplemental Figure 1). This decline in transmission aligns with the execution of Kenya’s 2001 National Malaria Strategy, in which a sequential scale-up of long-lasting insecticide-treated bed net (LLIN) use was implemented via social marketing in 2002, clinic-based distribution in 2004, and mass free LLIN campaigns in 2006.^60^ Because the introduction of AL as a designated first-line treatment for uncomplicated malaria and the stabilization of lower transmission both occurred around 2006, these factors are difficult to decouple. However, the authors suggest that they were mutually reinforcing and that the shift to sustained lower transmission likely exacerbated drug selection as waning population immunity led to a higher proportion of symptomatic cases. This likely increased treatment-seeking behavior and concentrated selective pressure during the subsequent period of sustained low transmission, potentially strengthening the impact of drug policy changes on the region’s *P. falciparum* genomic landscape.

The present study is limited by its retrospective nature and nonuniform sampling density across eras. The small sample size in certain years also limits fine-scale temporal analysis at several time points. Importantly, the study was not designed to independently analyze the impact of transmission intensity, and thus, the authors relied on secondary epidemiological data to contextualize the genomic findings. Furthermore, because western Kenya represents a higher-burden region relative to the rest of the country, the study results may not reflect the landscape of lower-transmission areas, which often favor resistance emergence. Molecular inversion probes offer a relatively low sensitivity for very low-parasite density infections and thus may not always sufficiently capture minority alleles. The authors also did not examine other complex factors, such as human movement across borders. Finally, although they correlate genomic shifts with policy milestones, they cannot account for individual-level drug exposure.

## CONCLUSION

Despite these limitations, the study analysis provides a comprehensive temporal record of the genomic shifts that have shaped the trajectory of *P. falciparum* antimalarial susceptibility in Kenya over the last two decades, offering vital evidence for the optimization of current treatment protocols and the strategic design of future antimalarial policies. Continued molecular surveillance is necessary to monitor emerging antimalarial drug resistance in Kenya, and efforts should be made to collect sufficient samples across diverse transmission and treatment-availability settings. Rapid molecular surveillance should be integrated with therapeutic efficacy studies to quantify the phenotypic impact of emerging markers, provide a more comprehensive and timely understanding of drug resistance evolution, and inform future antimalarial policy changes. This work is particularly important considering the recent detection of validated ART-R-associated K13 mutations in Kenya and surrounding countries. As ART-R expands regionally and ACT partner drug and preventative treatment resistance trends continue to evolve, enhanced genomic surveillance will be critical for guiding adaptive treatment policies and sustaining malaria control efforts.

## Supplemental Materials

10.4269/ajtmh.25-0513 Supplemental Materials
