# Supplementary material for: Two Decades of Molecular Surveillance in Kenya Reveal Shifting Plasmodium falciparum Drug Resistance Mutations Linked to Frontline Drug Changes
Source: Am J Trop Med Hyg. 2026 Nov 6:tpmd250513. Online ahead of print. doi: 10.4269/ajtmh.25-0513 (PMC13770347; doi:10.4269/ajtmh.25-0513)
Supplement: Supplementary file 1 [file tpmd250513.SD1.pdf]

Supplementary Materials

Supplementary Tables

Collection Year	Number of Sites	Total Samples
1998	1	4
2000	2	16
2002	1	6
2003	2	5
2005	3	21
2006	1	1
2008	3	38
2009	3	24
2010	3	41
2011	2	26
2012	1	1
2013	2	33
2014	3	84
2015	2	106
2016	3	23
2018	5	30
2019	5	102
2020	5	26
2021	6	24
PRE-ACT	2	14

Supplementary Table S1. Sample collection summary table. Annual counts of collected samples that underwent successful sequencing are presented. For historical samples labeled "PRE-ACT," exact calendar year metadata was unavailable, but documentation confirmed that the collection predated 2006.

**Abbreviation**	**Full Header Description**
Collection Year	The specific calendar year in which the biological samples were collected from the field sites.
Number of Sites	The total number of distinct geographic collection sites sampled within that specific year.
Total Samples	The overall count of collected samples from that year that were successfully sequenced and included in analysis.

Header Key for Table S1

Gene	Mutation	Pre-ACT (1998-2005)	ACT Transition (2006-2010)	Established ACT (2011-2021)	p-adj (A vs B)	p-adj (A vs C)	p-adj (B vs C)
K13	189T	52.4% (n=58)	18.2% (n=50)	27.0% (n=231)	0.067	0.072	0.446
K13	255K	6.3% (n=64)	6.0% (n=82)	9.3% (n=310)	1	1	1
K13	258M	2.0% (n=64)	4.6% (n=82)	4.9% (n=310)	0.516	0.516	0.578
K13	271H	5.0% (n=64)	2.4% (n=74)	3.9% (n=297)	1	1	1
K13	539T	0.0% (n=62)	0.0% (n=74)	0.0% (n=290)	NA	NA	NA
K13	561H	0.0% (n=60)	0.0% (n=54)	0.0% (n=260)	NA	NA	NA
K13	578S	0.9% (n=62)	5.3% (n=74)	1.4% (n=280)	0.598	0.671	0.598
K13	580Y	0.0% (n=62)	0.0% (n=74)	0.0% (n=280)	NA	NA	NA
UBP1	1525E	12.3% (n=39)	19.3% (n=48)	23.5% (n=181)	1	1	1
AP2MU	160N	47.7% (n=61)	39.3% (n=54)	19.0% (n=262)	0.548	0.015	0.111
ATP6	431K	27.5% (n=64)	29.1% (n=74)	25.9% (n=298)	1	1	1

Supplementary Table S2. Prevalence of *Plasmodium falciparum* K13, UBP1, AP2MU, ATP6 mutations across antimalarial policy eras in Kenya (1998–2021). Prevalence values are presented as percentages (%) with the number of mutant alleles (*m*) over the total number of successfully genotyped samples (*n*) in parentheses. The study period is divided into three distinct drug policy eras: Pre-ACT Rollout (1998–2005), ACT Transition (2006–2010), and Established ACT (2011–2021). Statistical significance for changes in prevalence between eras was determined using pairwise Wilcoxon rank-sum tests with *p*-values adjusted for multiple comparisons using the Holm-Bonferroni method.

Gene	Mutation	Pre-ACT (1998-2005)	ACT Transition (2006-2010)	Established ACT (2011-2021)	p-adj (A vs B)	p-adj (A vs C)	p-adj (B vs C)
MDR1	N86	52.3% (n=65)	57.5% (n=81)	100.0% (n=315)	0.619	< 0.001	< 0.001
MDR1	184F	41.3% (n=65)	36.8% (n=85)	57.5% (n=317)	0.523	0.239	0.157
MDR1	D1246	64.0% (n=62)	61.8% (n=70)	97.9% (n=269)	1	< 0.001	< 0.001

Supplementary Table S3. Prevalence of *Plasmodium falciparum* MDR1 mutations across antimalarial policy eras in Kenya (1998–2021). Prevalence values are presented as percentages (%) with the number of mutant alleles (*m*) over the total number of successfully genotyped samples (*n*) in parentheses. The study period is divided into three distinct drug policy eras: Pre-ACT Rollout (1998–2005), ACT Transition (2006–2010), and Established ACT (2011–2021). Statistical significance for changes in prevalence between eras was determined using pairwise Wilcoxon rank-sum tests with *p*-values adjusted for multiple comparisons using the Holm-Bonferroni method.

Gene	Mutation	Pre-ACT (1998-2005)	ACT Transition (2006-2010)	Established ACT (2011-2021)	p-adj (A vs B)	p-adj (A vs C)	p-adj (B vs C)
CRT	24Y	10.5% (n=61)	6.0% (n=79)	19.2% (n=290)	0.622	0.622	0.391
CRT	72S	0.0% (n=64)	0.0% (n=76)	0.0% (n=289)	NA	NA	NA
CRT	73L	0.0% (n=64)	0.0% (n=76)	0.2% (n=289)	NA	1	1
CRT	74I	91.1% (n=64)	78.2% (n=76)	9.7% (n=289)	0.204	< 0.001	< 0.001
CRT	75E	91.1% (n=64)	78.2% (n=76)	9.7% (n=289)	0.204	< 0.001	< 0.001
CRT	76T	91.1% (n=64)	78.2% (n=76)	9.7% (n=289)	0.204	< 0.001	< 0.001
CRT	220S	90.5% (n=57)	85.2% (n=52)	11.4% (n=236)	0.761	< 0.001	< 0.001
CRT	271E	93.4% (n=57)	81.1% (n=48)	8.4% (n=238)	0.424	< 0.001	< 0.001
CRT	326S	25.3% (n=60)	10.0% (n=64)	4.5% (n=289)	0.038	< 0.001	0.909
CRT	371I	91.7% (n=60)	75.1% (n=59)	11.6% (n=260)	0.123	< 0.001	< 0.001

Supplementary Table S4. Prevalence of *Plasmodium falciparum* CRT mutations across antimalarial policy eras in Kenya (1998–2021). Prevalence values are presented as percentages (%) with the number of mutant alleles (*m*) over the total number of successfully genotyped samples (*n*) in parentheses. The study period is divided into three distinct drug policy eras: Pre-ACT Rollout (1998–2005), ACT Transition (2006–2010), and Established ACT (2011–2021). Statistical significance for changes in prevalence between eras was determined using pairwise Wilcoxon rank-sum tests with *p*-values adjusted for multiple comparisons using the Holm-Bonferroni method.

Gene	Mutation	Pre-ACT (1998-2005)	ACT Transition (2006-2010)	Established ACT (2011-2021)	p-adj (A vs B)	p-adj (A vs C)	p-adj (B vs C)
DHFR-TS	51I	90.7% (n=63)	96.5% (n=76)	99.6% (n=281)	0.929	0.035	0.035
DHFR-TS	59R	83.2% (n=63)	91.9% (n=76)	98.4% (n=281)	0.549	0.012	0.549
DHFR-TS	108N	97.7% (n=61)	98.3% (n=73)	99.8% (n=277)	1	1	1
DHFR-TS	164L	1.1% (n=58)	3.0% (n=70)	7.1% (n=268)	0.944	0.845	0.845

Supplementary Table S5. Prevalence of *Plasmodium falciparum* DHFR-TS mutations across antimalarial policy eras in Kenya (1998–2021). Prevalence values are presented as percentages (%) with the number of mutant alleles (*m*) over the total number of successfully genotyped samples (*n*) in parentheses. The study period is divided into three distinct drug policy eras: Pre-ACT Rollout (1998–2005), ACT Transition (2006–2010), and Established ACT (2011–2021). Statistical significance for changes in prevalence between eras was determined using pairwise Wilcoxon rank-sum tests with *p*-values adjusted for multiple comparisons using the Holm-Bonferroni method.

Gene	Mutation	Pre-ACT (1998-2005)	ACT Transition (2006-2010)	Established ACT (2011-2021)	p-adj (A vs B)	p-adj (A vs C)	p-adj (B vs C)
DHPS	436A	19.1% (n=50)	2.0% (n=73)	0.0% (n=240)	0.280	0.006	0.147
DHPS	436H	0.0% (n=43)	3.3% (n=75)	20.3% (n=280)	0.167	0.005	0.022
DHPS	437G	75.9% (n=60)	98.3% (n=74)	96.2% (n=247)	0.115	0.030	0.859
DHPS	540E	69.8% (n=64)	98.0% (n=76)	96.1% (n=288)	0.047	0.011	0.725
DHPS	581G	0.0% (n=65)	0.0% (n=80)	2.1% (n=327)	NA	0.500	0.500
DHPS	613S	5.1% (n=64)	0.0% (n=75)	0.0% (n=317)	0.093	0.004	NA

Supplementary Table S6. Prevalence of *Plasmodium falciparum* DHPS mutations across antimalarial policy eras in Kenya (1998–2021). Prevalence values are presented as percentages (%) with the number of mutant alleles (*m*) over the total number of successfully genotyped samples (*n*) in parentheses. The study period is divided into three distinct drug policy eras: Pre-ACT Rollout (1998–2005), ACT Transition (2006–2010), and Established ACT (2011–2021). Statistical significance for changes in prevalence between eras was determined using pairwise Wilcoxon rank-sum tests with *p*-values adjusted for multiple comparisons using the Holm-Bonferroni method.

**Abbreviation**	**Full Header Description**
Gene	The specific *Plasmodium falciparum* genetic locus under investigation.
Mutation	Specific genetic variant defined by the amino acid substitution and its corresponding codon position.
Pre-ACT (1998-2005)	Baseline historical period prior to the official rollout of artemisinin-based combination therapy (ACT) (1998–2005). Values display mutant allele prevalence (%) alongside the total number of successfully genotyped samples (*n*) at the locus.
ACT Transition (2006-2010)	Intermediate epidemiological period marking the phased introduction and adoption of ACT (2006–2010). Values display mutant allele prevalence (%) alongside the total number of successfully genotyped samples (*n*) at the locus.
Established ACT (2011-2021)	Period of sustained, widespread stabilization and implementation of ACT policy (2011–2021). Values display mutant allele prevalence (%) alongside the total number of successfully genotyped samples (*n*) at the locus.
p-adj (A vs B)	Holm-Bonferroni adjusted *p*-value comparing mutant prevalence between the Pre-ACT and ACT Transition eras.
p-adj (A vs C)	Holm-Bonferroni adjusted *p*-value comparing mutant prevalence between the Pre-ACT and Established ACT eras.
p-adj (B vs C)	Holm-Bonferroni adjusted *p*-value comparing mutant prevalence between the ACT Transition and Established ACT eras.

Header Key for Tables S2-S6

Supplementary Figures

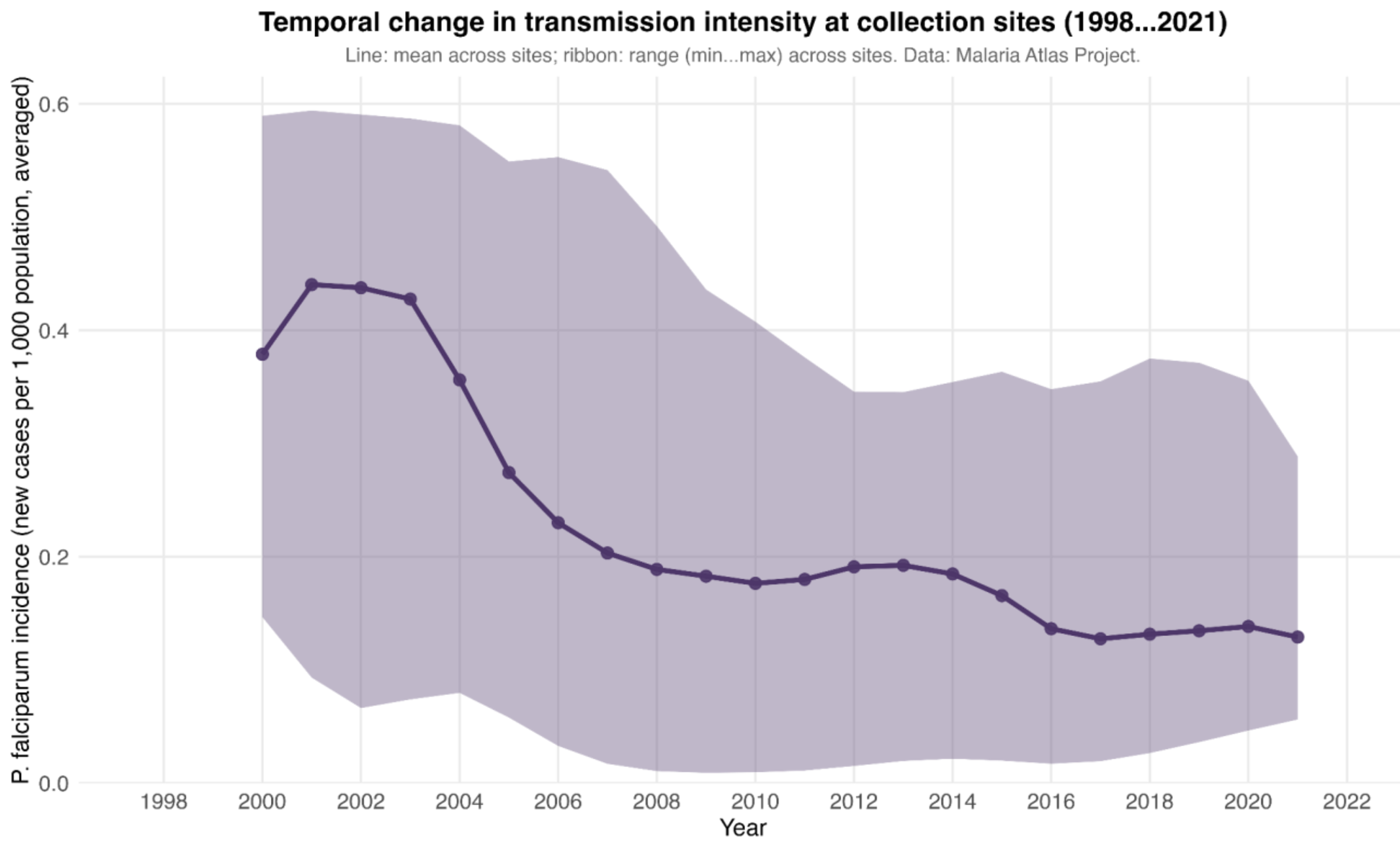

Supplementary Figure S1: Temporal line plot of mean malaria incidence rates across our 11 study sites (2000–2021) using data obtained from the Malaria Atlas Project (MAP).

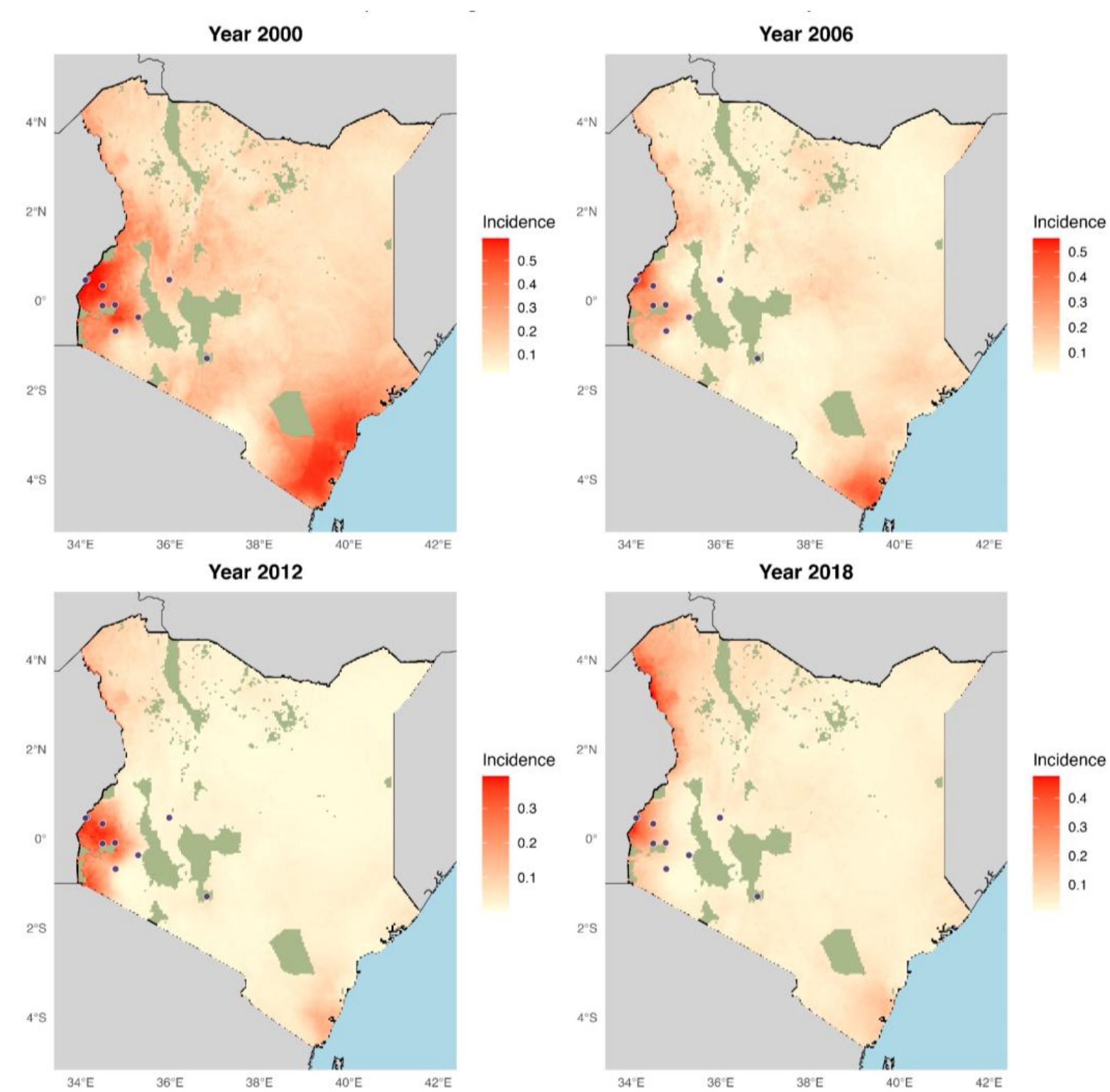

Supplementary Figure S2: 4-panel geospatial choropleth of yearly mean malaria incidence rates across Kenya in 2000, 2006, 2012, and 2018 using data obtained from the Malaria Atlas Project (MAP).

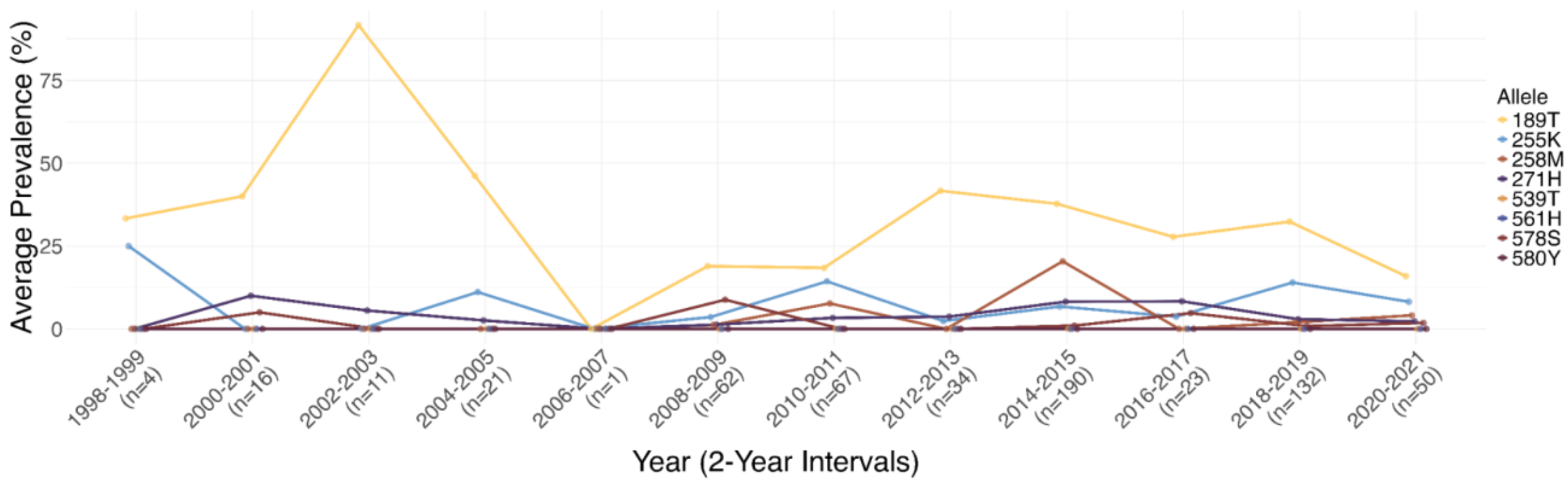

Supplementary Figure S3: Temporal trends in nonsynonymous *pfk13* allele prevalences identified in this study shown as 2-year moving averages. Annual sample sizes for the total dataset are provided in the x-axis labels.
